# Maternal Impressions

**Published:** 1877-09-20

**Authors:** Geo. A. Dyer

**Affiliations:** Washington, Ind.


### MATERNAL IMPRESSIONS.

BY GEO. A. DYER, M.D., WASHINGTON, IND.

Last December, I read, in the *Detroit Review of Medicine and Pharmacy*, the very interesting address of Dr. Waddell, delivered before the Toledo (O.) Medical Association, on the above subject. Many persons believe that, at a certain stage of gestation, impressions made on the mother have power over the fœtus in utero to change its conformation, and cause deformity of some character. From time immemorial, this opinion has obtained among observant women, and cases have presented themselves to physicians in such shapes, and with such explanations, that one can scarcely doubt the fact.

To myself have happened several such cases. So far as deformities are concerned, it is a well known fact that many are hereditary. I knew a colored woman who had a well formed fifth finger (the little finger) on one hand; her mother had the same mark, and she herself was delivered of two children who had the same. Not long ago I attended a lady whose child had an abortive extra little finger about a half inch long, hanging by a thread-like pedicle, fully an inch and a half long, which latter I ligated close to the finger with a very fine silken cord, and it fell off in a few days. The mother told me that her husband was born with the same kind of finger, and it was removed in infancy by the same method.

These deformities or marks often happen, and therefore do not seem strange. But as to impressions, why should a child be born of a woman with a perfect arm but an abortive fore-arm, and she herself should say that she had seen a man, in early gestation with this child, who had the same deformity, and she believes that the sympathy she felt for the stranger, and the impression of his deformity, caused her child to be afflicted with the same mark?

Several years ago, Barnum's show came to this place, and with the show were the idiots known as the "wild Australian children." A Mrs. S., a well developed and pretty young woman, lately married, found herself pregnant, who, though advised by her lady friends not to go to the show, still had such a powerful temptation and desire to go, that she could not and did not resist. She went. She was seen to be horror-stricken at the appearance of these wild children, and rivited, as it were, to the place. Some five or six months after, I was called to accouch her. She had a tedious labor, but in a few hours was delivered of the strangest being I ever saw. The frontal, parietal and occipital bones, and the lateral and spinous processes of the vertebræ, were all wanting. There was no appearance of brain or spinal cord, but a bloody membrane covering the floor of the brain and the sulci of the vertebræ, their whole length, and the child resembled the Australian children in this, that from the supra-orbital ridge there was no calvaria whatever; and yet the child was well grown and well developed otherwise, a boy, and animal life was present not long before it was expelled. It weighed some six pounds. This is yet in Washington, Ind., in alcohol.

Again, in December, 1875, I accouched a Mrs. S., multipara. She had a tedious labor, and when the waters broke, the infant fluttered in the womb like a fish out of water, but it died there, and was still-born. When born, I discovered it was deformed. The mother suspected all was not right, and asked if it was not deformed, and when told it was, said she could easily account for its appearance. Early in gestation, after the middle of the night, she was awake; the door was open, and a person like a woman with a deformed head walked in at the door, whom, when she saw, she screamed and sat up in her bed, terrified with fright. Her husband saw nothing; he however closed the door. She slept no more that morning. Now, the infant's head (which she examined) was set so close to the body that it appeared to have no neck, a flattened calvaria, ears like a dog, and its mouth was like a hog's snout. She insisted that it had the same appearance of the figure she saw, and she also believed that the deformity was from the impression made upon her in the early stage of gestation. Well for the child to have been still-born.

Other cases of this kind have been told to me by physicians and mid-wives here.

These both seem to be well-marked cases of arrest of development and consequent deformity. Did they so occur from impressions made on the mother? is the grave question.

[There is manifestly, of late years, a growing tendency on the part of medical men to give credence to the prevailing sentimeni of the people on this subject of maternal impression. Certainly very strong coincidences occur. The subject is important and worthy of investigation. ED]

